# Loose Binding of the DF Axis with the A_3_B_3_ Complex Stimulates the Initial Activity of *Enterococcus* *hirae* V_1_-ATPase

**DOI:** 10.1371/journal.pone.0074291

**Published:** 2013-09-13

**Authors:** Md. Jahangir Alam, Satoshi Arai, Shinya Saijo, Kano Suzuki, Kenji Mizutani, Yoshiko Ishizuka-Katsura, Noboru Ohsawa, Takaho Terada, Mikako Shirouzu, Shigeyuki Yokoyama, So Iwata, Yoshimi Kakinuma, Ichiro Yamato, Takeshi Murata

**Affiliations:** 1 Department of Biological Science and Technology, Tokyo University of Science, Chiba, Japan; 2 Department of Genetic Engineering and Biotechnology, School of Life Sciences, Shahjalal University of Science and Technology, Sylhet, Bangladesh; 3 Department of Chemistry, Graduate School of Science, Chiba University, Chiba, Japan; 4 RIKEN SPring-8 Center, Hyogo, Japan; 5 Structural Biology Research Center, Photon Factory, Institute of Materials Structure Science, High Energy Accelerator Research Organization (KEK), Ibaraki, Japan; 6 RIKEN Systems and Structural Biology Center, Yokohama, Japan; 7 Department of Cell Biology, Faculty of Medicine, Kyoto University, Kyoto, Japan; 8 Laboratory of Molecular Physiology and Genetics, Faculty of Agriculture, Ehime University, Ehime, Japan; 9 JST, PRESTO, Chiba, Japan; University of Strathclyde, United Kingdom

## Abstract

Vacuolar ATPases (V-ATPases) function as proton pumps in various cellular membrane systems. The hydrophilic V_1_ portion of the V-ATPase is a rotary motor, in which a central-axis DF complex rotates inside a hexagonally arranged catalytic A_3_B_3_ complex by using ATP hydrolysis energy. We have previously reported crystal structures of *Enterococcus* *hirae* V-ATPase A_3_B_3_ and A_3_B_3_DF (V_1_) complexes; the result suggested that the DF axis induces structural changes in the A_3_B_3_ complex through extensive protein-protein interactions. In this study, we mutated 10 residues at the interface between A_3_B_3_ and DF complexes and examined the ATPase activities of the mutated V_1_ complexes as well as the binding affinities between the mutated A_3_B_3_ and DF complexes. Surprisingly, several V_1_ mutants showed higher initial ATPase activities than wild-type V_1_-ATPase, whereas these mutated A_3_B_3_ and DF complexes showed decreased binding affinities for each other. However, the high ATP hydrolysis activities of the mutants decreased faster over time than the activity of the wild-type V_1_ complex, suggesting that the mutants were unstable in the reaction because the mutant A_3_B_3_ and DF complexes bound each other more weakly. These findings suggest that strong interaction between the DF complex and A_3_B_3_ complex lowers ATPase activity, but also that the tight binding is responsible for the stable ATPase activity of the complex.

## Introduction

Vacuolar ATPase (V-ATPase) functions as a proton pump in acidic organelles and plasma membranes of eukaryotic cells and bacteria [1,2]. The acidic environment is essential for processes such as receptor-mediated endocytosis, intracellular targeting of lysosomal enzymes, protein processing, and degradation [1]. V-ATPase contains a globular catalytic domain, V_1_, that hydrolyses ATP, and this domain is attached by central and peripheral stalks to an integral membrane domain, V_o_, that pumps ions across the membrane. ATP hydrolysis triggers the rotation of the central stalk and an attached membrane ring of hydrophobic subunits. Ions are pumped through a channel formed at the interface between the rotating ring and a static membrane component, which is linked to the outside of the V_1_ domain by the peripheral stalks [1].

**Figure 1:**
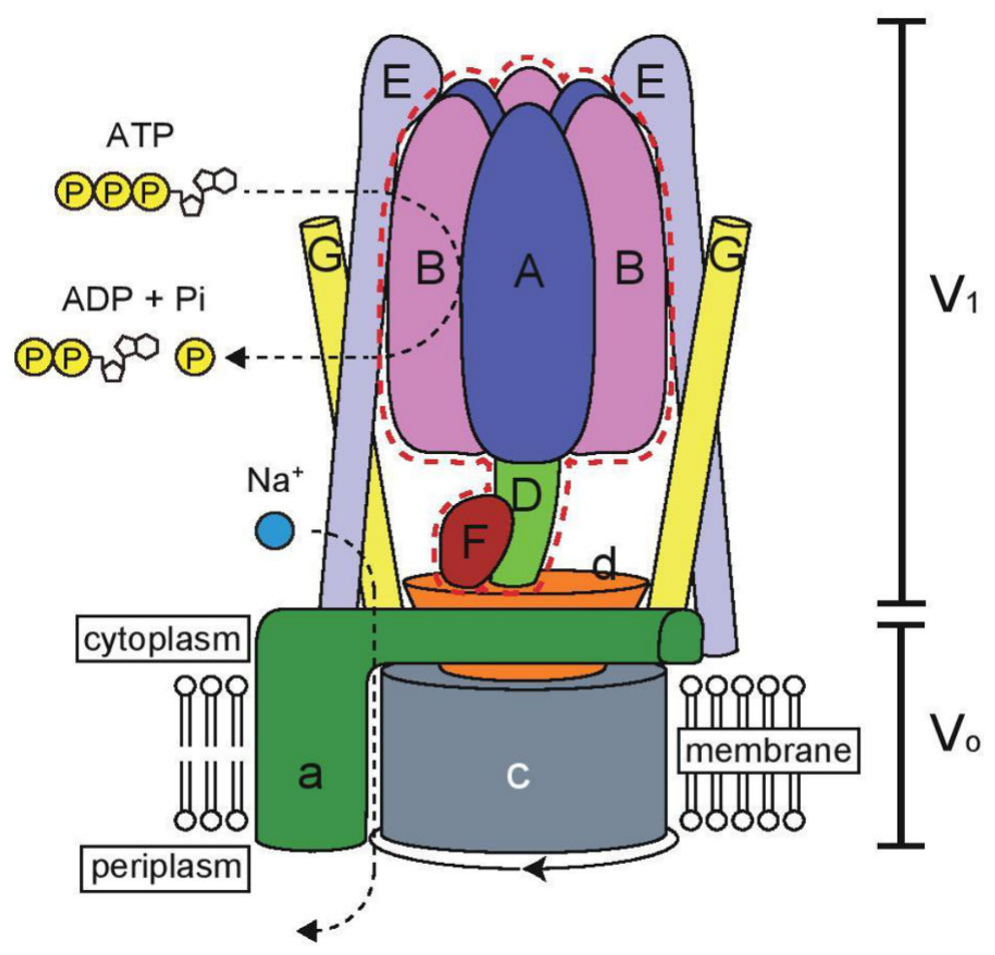
Schematic model of *E* *. hirae* V-ATPase (adapted from [15] and [16]). The V_1_ domain of V-ATPase is composed of a hexameric arrangement of alternating A and B subunits responsible for ATP binding and hydrolysis; it also contains the DF subunits (shown by a dotted red line), the focus of this study. The V_o_ domain of V-ATPase comprises an a subunit and an attached membrane c ring. The V_1_ and V_o_ domains are connected by a central stalk, which is composed of D, F, and d subunits, and 2 peripheral stalks assembled from the E and G subunits of V_1_. ATP hydrolysis induces the rotation of the central axis (DFd complex) together with the c ring, which causes Na^+^ to be pumped through the channel at the interface between the c ring and the a subunit.

**Figure 2:**
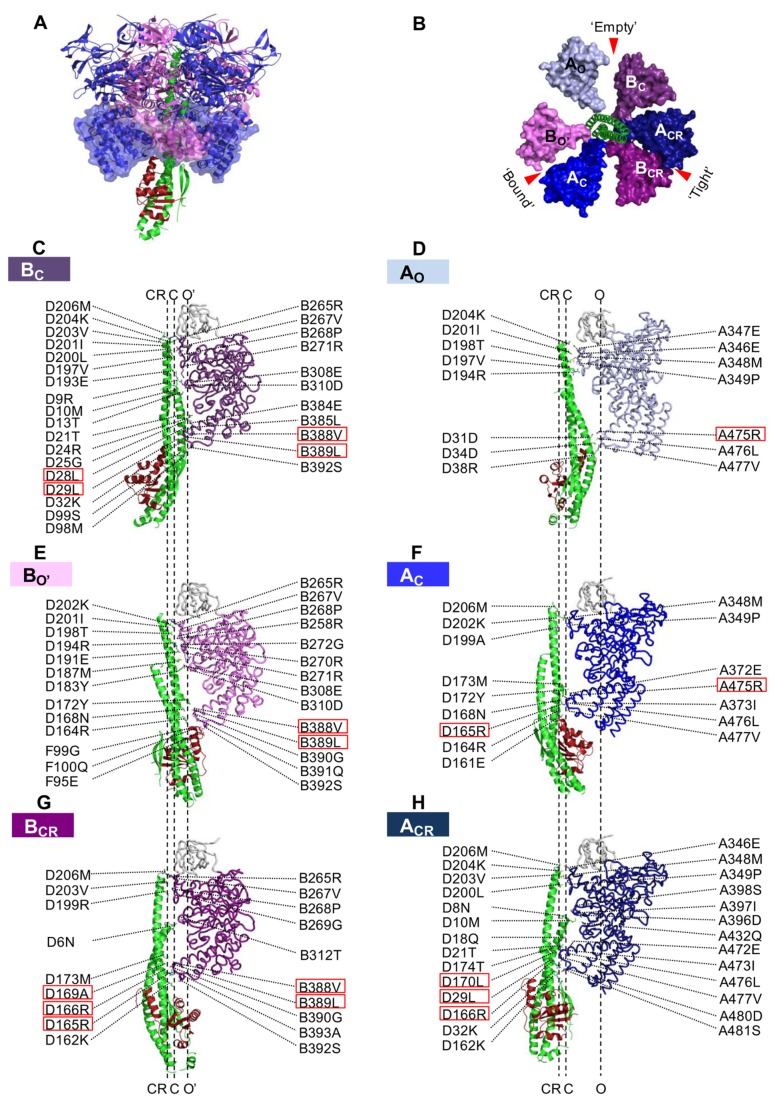
Structure of nucleotide-free A_3_B_3_DF and protein-protein interactions between the A_3_B_3_ and DF complexes in *E* *. hirae* V_1_-ATPase. *A*, Side view of the nucleotide-free A _3_B_3_DF (V_1_) complex of *E* *. hirae* . D and F subunits are represented in green and red, respectively. *B*, Top view of the C-terminal domain of *E* *. hirae* V_1_-ATPase. Empty (O and O′), bound (C), and tight (CR) conformations of *E* *. hirae* A and B subunits are shown in light, dark, and darker colors, respectively. Red arrows indicate nucleotide-binding sites. The side-view ribbon representations show the residues of B_C_ (*C*), A_O_ (*D*), B_O’_ (*E*), A_C_ (*F*), B_CR_ (*G*), and A_CR_ (*H*) that interact with the residues of the DF complex in *E* *. hirae* V_1_-ATPase. The sticks represent the residues with a buried surface area > 10 Å^2^, as calculated by PDBePISA (http://pdbe.org/pisa/). The residues in the red boxes were mutated in this study.

V-ATPases are found in bacteria such as *Thermus thermophilus* and *Enterococcus* *hirae* . *T. thermophilus* V-ATPase functions physiologically as an ATP synthase [3], whereas the *E* *. hirae* V-ATPase, which transports Na^+^ or Li^+^ instead of H^+^ [4-8], is not an ATP synthase and acts instead as a primary ion pump like eukaryotic V-ATPases. The amino acid sequences and subunit structures of *E* *. hirae* V-ATPase are more similar to those of eukaryotic V-ATPases than to those of ATP synthases of the F- and V-type ATPase families. The enzyme has 9 subunits whose amino acid sequences are homologous to sequences from corresponding subunits of eukaryotic V-ATPases [9-12]. The core of *E* *. hirae* V_1_ domain is composed of a hexameric arrangement of alternating A and B subunits that are responsible for ATP binding and hydrolysis [13]. The V_o_ domain, which uses rotational energy to drive Na^+^ translocation, is composed of oligomers of the 16-kDa c subunits and an a subunit [7,8]. The V_1_ and V_o_ domains are connected by a central stalk, composed of the D, F, and d subunits, and 2 peripheral stalks comprising the E and G subunits of V_1_ (Figure 1) [11, 13]. ATP hydrolysis induces the rotation of the central axis (DFd complex) and the attached membrane c ring, which results in ions being pumped through a channel at the interface between the c ring and the a subunit [6]. Recently, we purified the A_3_B_3_ and DF complexes and reconstituted the V_1_-ATPase using the 2 complexes [14] and we determined the crystal structures of the DF, A_3_B_3_, and A_3_B_3_DF complexes [15,16]. This structural information suggests that the DF complex binds the A_3_B_3_ complex tightly through 19 polar interactions and 101 nonpolar (van der Waals) interactions; through these interactions, the DF complex induces conformational changes in the A_3_B_3_ complex (Figure 2C-H), and ATP hydrolysis appears to be stimulated by the approach of a conserved arginine residue (arginine finger) [15].

The general architectures and mode of actions of V-ATPase and F-ATPase are similar [17]. The isolated F_1_ domain, like the V_1_ domain, is composed of α_3_β_3_γ(δε) and hydrolyses ATP [18], with the γ-subunit serving as the rotation axis. The contact between γ and α_3_β_3_, especially at the DELSEED region, has been examined extensively by mutagenesis to elucidate the rotation mechanism [19-22].

To date, 120 polar and nonpolar (van der Waals) interactions have been identified between the DF and A_3_B_3_ complexes (Figure 2C-H) [15]. In this study, we chose 10 residues for creating site-directed mutants of A, B, and D subunits (1 mutation in A, 2 in B, and 7 in D subunits (Figure 2, residues in red boxes)). These residues located at the interface between the D subunit and the C-terminal domain of A and/or B subunits [15] (and likely, correspond to the DELSEED region in F_1_) were selected for mutation, taking into account the conservation, importance, and location of each amino acid. All 10 residues appeared to interact with each of the other 2 subunits in at least 1 of their 3 conformational states (A_CR_-B_CR_ pair, “tight” form; A_C_-B_O_ pair, “bound” form; and A_O_-B_C_ pair, “empty” form; Figure 2B). We reconstituted mutant V_1_-domains, and measured the ATPase activities and binding affinities of the mutant A_3_B_3_ for the DF axis (wild-type or mutant DF). It has been suggested that ATPases showing higher activities have lower subunit-subunit binding affinities and vice versa [23,24]. In this study, we determined the critical reciprocal relationship between ATPase activities and subunit-subunit binding affinities by using mutant V_1_-ATPases, suggesting that loose binding of the DF axis with the rotary A_3_B_3_ ring enhances the activity of V_1_-ATPase. A similar reciprocal relationship has been observed recently in *Escherichia coli* F-ATPase [25]. Our results also suggest that tight binding of the DF complex with the A_3_B_3_ complex lowers ATPase activity but that the strong interaction ensures stable ATPase activity in the A_3_B_3_DF complex.

## Materials and Methods

### Expression and Purification of the A_3_B_3_ Complex

Synthesized DNA fragments corresponding to the *A* and *B* genes with optimal codon usage for an *Escherichia coli* expression system were cloned into the plasmid vector pET23d [14]. Mutant A and B subunits were constructed using the wild-type *A* and *B* genes, respectively, in the plasmids as template for PCR mutagenesis. A and B subunits were expressed separately in *E. coli* BL21 (DE3) grown in a modified-Davis Mingioli-Casamino Acid (m-DM-CA) medium [26] at 30°C, and the 2 subunits were then purified and reconstituted as described previously [14]. Briefly, the purified A and B subunits (3.4 and 2.7 mg of A and B subunits, respectively, in a 1: 1 molar ratio) were mixed and incubated for 1 h on ice in buffer A (20 mM MES-Tris, pH 6.5; 50 mM KCl; 10% glycerol; 5 mM MgSO_4_; 0.1 mM DTT) in the presence of 2 mM ATP and were then concentrated to 100 µL by ultrafiltration using Amicon Ultra-4 30K filters (Millipore Corporation, USA). Next, 4 mL of buffer A containing ATP was added to dilute the protein solution, and the solution was concentrated again to 100 µL. This dilution/concentration process was then repeated thrice without adding ATP, and the A_3_B_3_ heterohexamer was purified finally using a Superose 6 pg column (500 × 16 mm ID) (GE Healthcare). The formation of the complex was confirmed by using basic native polyacrylamide gel electrophoresis (PAGE) as described [14].

### Expression and Purification of the DF Complex

An *E. coli* cell-free protein expression system [27] was used to synthesize the DF complex by using plasmids carrying D and F subunit genes. More than 0.5 mg of the complex was synthesized with this system in 1 mL of the reaction solution in the presence of 3 µg of plasmid [27] and the expressed proteins were purified as described previously [11]. The D subunit was mutated using the QuikChange site-directed mutagenesis kit (Agilent Technologies).

### Reconstitution of the V_1_ (A_3_B_3_DF) Complex

The catalytic V_1_ (A_3_B_3_DF) complex was reconstituted from purified A_3_B_3_ and DF complexes as described in earlier studies [14,15]: Briefly, purified A_3_B_3_ and DF complexes were mixed in a 1: 5 molar ratio and incubated on ice for 1 h, and complex formation was confirmed by using basic native-PAGE [14].

### Measurement of ATPase Activity of Mutant A_3_B_3_DF Complexes

ATPase activities of the reconstituted A_3_B_3_DF complexes were measured using an ATP regenerating system [28]. The reaction mixture contained various concentrations of ATP, 2.5 mM phosphoenolpyruvate, 50 µg/mL pyruvate kinase, 50 µg/mL lactate dehydrogenase, and 0.2 mM β-NADH (dipotassium salt) in 1 mL of buffer B (25 mM MES-Tris, pH 6.5; 4 mM MgSO_4_; and 10% glycerol). Reactions were initiated by adding 1–2 µg of proteins, and ATP hydrolysis rates (at 25°C) were determined in terms of the rate of NADH oxidation, which was measured as a decrease in absorbance at 340 nm. Specific activities were calculated as units/mg proteins, with 1 unit of ATPase activity being defined as hydrolysis of 1 µmol ATP/min. Initial ATPase activity was calculated by measuring the specific activity during the first minute (starting from the 16^th^ second) after adding the protein. Because the measurement curve was concave even for wild-type V_1_, we concluded that the activity was not stable in the reaction mixture with ATP. Thus, the stability of reconstituted A_3_B_3_DF mutants was estimated using time-course experiments: ATPase activity was measured at 2-min intervals for 20 min. The measurements were repeated thrice and averaged, and the standard deviations were calculated. *K*_m_ and *V*_max_ were then calculated by fitting the average values as straight lines in Lineweaver-Burk plots.

### Measurement of Binding Affinity Using Surface Plasmon Resonance (SPR)

The binding affinity of the DF complex for the reconstituted A_3_B_3_ complex was measured using SPR analysis on a Biacore T100 instrument (GE Healthcare Bio-sciences, AB, Sweden) as described previously [15,16]. The Biacore Ni-NTA sensor chip (GE Healthcare Bio-sciences) was activated using 0.5 µM NiCl_2_ as per the manufacturer’s instructions. For analyses, we used His-tagged A_3_B_3_ as the ligand and DF as the analyte. His-tagged A_3_B_3_ was reconstituted using A subunits treated with TEV protease (tobacco etch virus protease) and His-tagged B subunits; the complex was reconstituted following protocols described above (in “Expression and purification of the A_3_B_3_ complex”). The reconstituted His-tagged A_3_B_3_ complex was immobilized on the sensor chip at a concentration of 35 µg/mL in running buffer (20 mM MES-Tris, pH 6.5; 150 mM NaCl; 50 µM EDTA-Na; 0.005% polyoxyethylene [20] sorbitol monolaurate) by passing the protein solution through the Biacore flow cell at a rate of 10 µL/min. A flow cell containing no immobilized protein served as the negative control. Several concentrations of the DF complex were prepared in running buffer and used as the analyte. The sensorgrams obtained were examined using the Biacore T100 evaluation software, and the equilibrium constant for dissociation (*K*_D_) was calculated using the Langmuir binding model (1:1 binding).

### Other Experimental Procedures

Protein concentrations were determined using the DC Protein Assay Kit (Bio-Rad Laboratories) with bovine serum albumin serving as the standard. Protein purification steps were evaluated using sodium dodecyl sulfate-PAGE (SDS-PAGE) [29] and samples were stained with Coomassie Brilliant Blue R-250. Restriction enzymes were purchased from Nippon Gene Japan, New England BioLabs Japan, and Wako Pure Chemical Industries Ltd. All other chemicals were of analytical grade and were obtained from Sigma-Aldrich Japan KK or Wako Pure Chemical Industries Ltd.

## Results and Discussion

### Properties of Central Axis D Mutants

**Figure 3:**
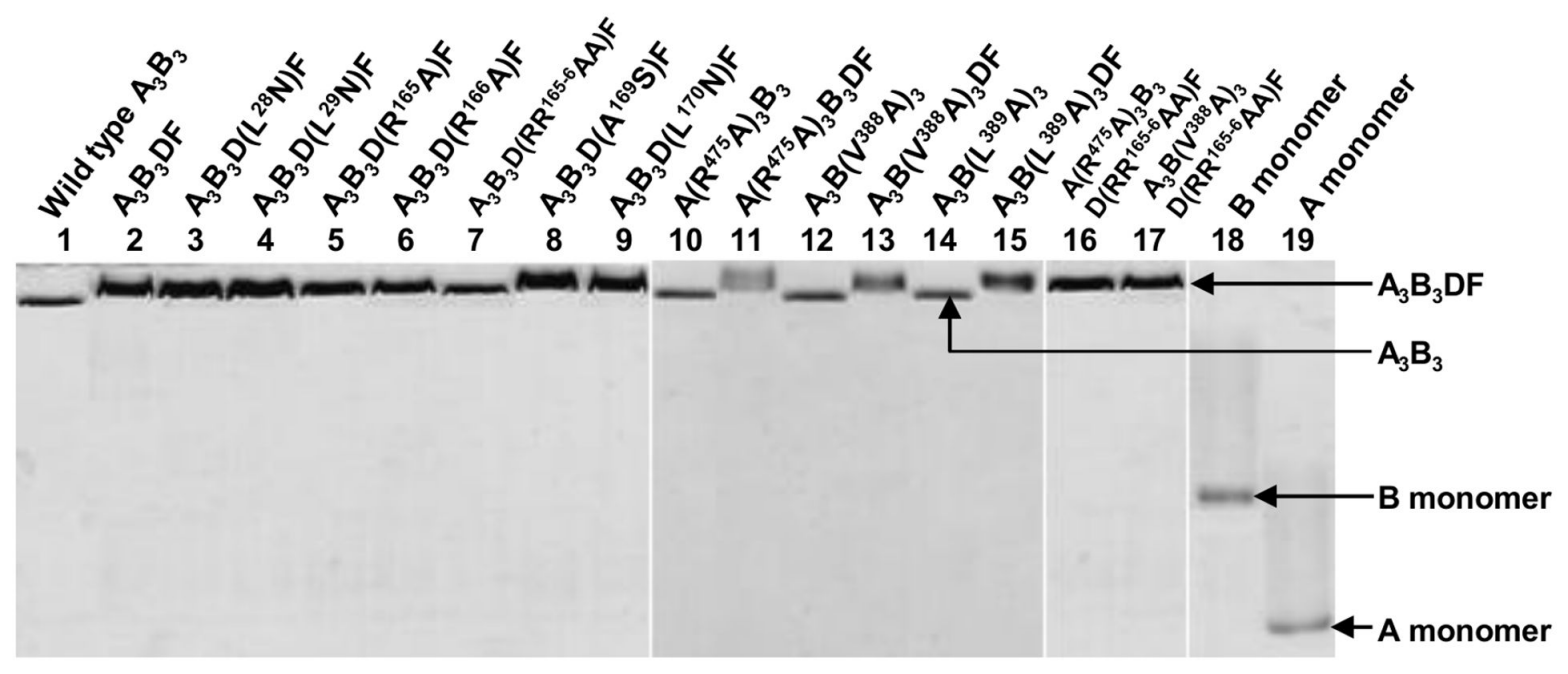
Basic native-PAGE patterns for the reconstitution of wild-type/mutant catalytic domains (V_1_ domains). Purified wild-type or mutant A _3_B_3_ and DF complexes were mixed in a 1: 5 molar ratio and incubated on ice for 1 h to reconstitute the catalytic domain A _3_B_3_DF, as described in Materials and Methods. Lanes 1, 10, 12, and 14: purified wild-type and mutant A _3_B_3_ complexes; lane 2: wild-type A _3_B_3_DF; lanes 3, 9, 11, 13, 15, 17: reconstituted mutant catalytic domains; and lanes 18 and 19: B and A monomers, respectively. Three micrograms of proteins were loaded in lanes 1, 9, 16, and 17, and 2 µg in lanes 10, 15, 18, and 19.

From the crystal structures of A_3_B_3_ and A_3_B_3_DF [15], we identified 120 polar and nonpolar (van der Waals) interactions between the DF complex and the A_3_B_3_ complex in the A_3_B_3_DF complex (Figure 2C-H). In this study, we selected 6 amino acids in the D subunit for mutation (L^28^, L^29^, R^165^, R^166^, A^169^, and L^170^); these residues are located at the binding interface between the A, B, and D subunits and reside close to the C-terminal domain of the A and/or B subunits of the *E* *. hirae* V_1_-ATPase [15]. We constructed D(L^28^N) F, D(L^29^N) F, D(R^165^A) F, D(R^166^A) F, D(A^169^S) F, and D(L^170^N) F mutants and reconstituted the corresponding mutant catalytic V_1_ domains using the wild-type A_3_B_3_ heterohexamer. As shown in Figure 3, all DF mutants reconstituted V_1_ domains to a similar extent as wild-type. Among the mutants, D(L^28^N) F, D(L^29^N) F, and D(L^170^N) F showed almost wild-type levels of initial ATPase activities and binding affinities, whereas D(A^169^S) F showed slightly lower initial ATPase activity and slightly higher binding affinity than the wild-type DF (Table 1).

**Table 1:** Summary of ATPase activities of V_1_ complexes containing wild-type A_3_B_3_ and mutant DF and their binding affinities measured using SPR assays.

**Protein**	**(%**) of initial specific activity^*^	(**%**) of ATPase activity after 15 minutes of the assay^**^	***K*_D_ (nM**)** (using wild-type A_3_B_3_ as ligand and mutant DF as analyte**)
**Wild-type A_3_B_3_DF**	100	44	1.6 ± 0.1
**A_3_B_3_D(L^28^N**)**F**	91	40	2.7 ± 0.2
**A_3_B_3_D(L^29^N**)**F**	103	19	5.0 ± 0.9
**A_3_B_3_D(R^165^A**)**F**	121	29	6.5 ± 1.7
**A_3_B_3_D(R^166^A**)**F**	179	31	7.3 ± 0.6
**A_3_B_3_D(RR^165-6^AA**)**F**	191	5	17.0 ± 0.1
**A_3_B_3_D(A^169^S**)**F**	82	73	0.9 ± 0.1
**A_3_B_3_D(L^170^N**)**F**	104	68	5.3 ± 0.5

ATPase activities of reconstituted A_3_B_3_DF mutants were analyzed as described in Materials and Methods, with assays being started by adding 1–2 µg protein, depending on the mutants. For SPR assays, DF heterodimers at various concentrations (analyte) were injected onto the sensor chip Ni-NTA surface with immobilized wild-type A_3_B_3_ heterohexamers, as described previously [15,16]. Reconstituted wild-type A_3_B_3_ heterohexamers and mutant DF heterodimer were diluted in running buffer; the experimental procedures used are described in Materials and Methods.

^*^“Initial specific activity” was calculated by measuring the specific activity during the first minute of the assay (starting from the 16^th^ second) after adding proteins. We calculated the percentage of the initial specific activity of the mutant V_1_ domains, considering the specific activity of the wild-type A_3_B_3_DF as 100%.

^**^“Activity after 15 min” was the specific ATPase activity measured at the 16^th^ min (from 15: 01 to 16.00 min) during the extended assay. These values were calculated considering the initial ATPase activity of each mutant as 100%.

**Figure 4:**
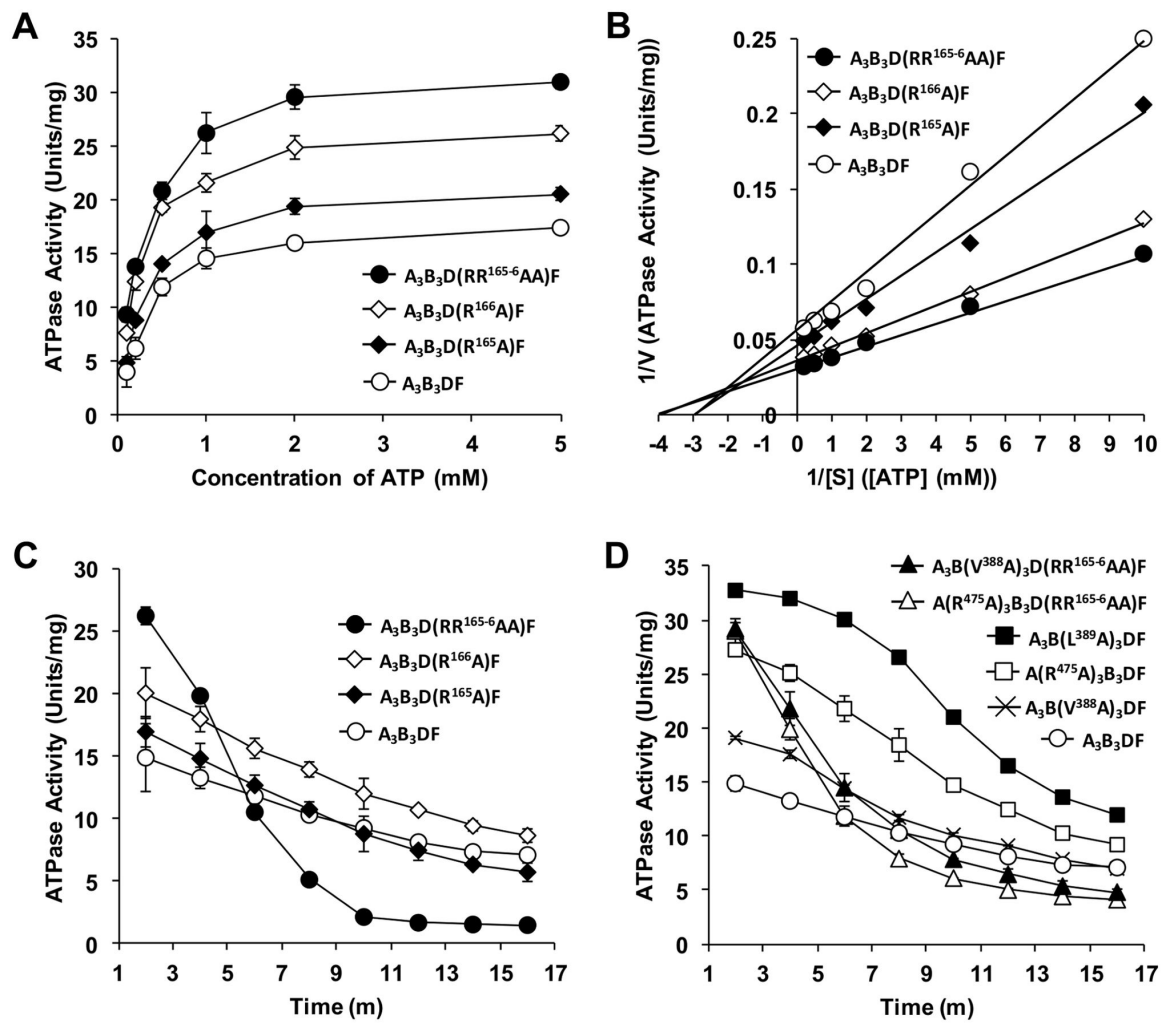
ATPase activities and their stability in mutant A_3_B_3_DF complexes of *E* *. hirae* V-ATPase. ATPase activities of the mutants were measured using an ATP regeneration system as described in Materials and Methods. *A*, ATPase activities of the central-axis D subunit mutants measured using various concentrations of ATP. *B*, Lineweaver-Burk plots of the ATPase activities from Figure 4A used for calculating *K*_m_ and *V*_max_ values for the D mutants. *C*-*D*, Stability of ATPase activities of mutant A _3_B_3_DF complexes. ATPase activities were measured in the presence of 1 mM ATP. *Filled* *circles*, A _3_B_3_D(RR^165-6^AA) F; *open* *diamonds*, A _3_B_3_D(R^166^ A) F; *filled* *diamonds*, A _3_B_3_D(R^165^ A) F; *filled* *triangles*, A _3_B(V^388^ A) _3_D(RR^165-6^AA) F; *open* *triangles*, A(R^475^ A) _3_B_3_D(RR^165-6^AA) F; *filled* *squares*, A _3_B(L^389^ A) _3_DF; *open* *squares*, A(R^475^ A) _3_B_3_DF; *open* *crosses*, A _3_B(V^388^ A) _3_DF; and *open* *circles*, wild-type A _3_B_3_DF.

In contrast to other mutants, D(R^165^A) F and D(R^166^A) F showed higher initial ATPase activities and lower binding affinities than wild-type DF (Figure 4A and Table 1): D(R^165^A) F and D(R^166^A) F had 1.2- and 1.8-times higher initial ATPase activities than the wild-type and lower binding affinities (*K*_D_ values) for the A_3_B_3_ heterohexamer, that is, 6.5 and 7.3 nM, respectively, than the wild-type (*K*_D,_ 1.6 nM) (Table 1). Considering the high initial specific activities of the mutant V_1_-ATPases and their lower binding affinities of these 2 single mutants, we constructed the double mutant D(RR^165-6^AA) F and reconstituted the corresponding catalytic domain with wild-type A_3_B_3_ heterohexamer (Figure 3). Surprisingly, D(RR^165-6^AA) F had almost 2-times higher initial ATPase activity (191%, Figure 3A and Table 1) than wild-type DF and also had considerably lower binding affinity (*K*_D_ = 17 nM, Table 1) for the A_3_B_3_ heterohexamer than wild-type DF. The ATPase activities of the 3 mutants, D(R^165^A) F, D(R^166^A) F, and D(RR^165-6^AA) F, had similar *K*_m_ values (0.33, 0.27, and 0.26 mM, respectively) and higher *V*_max_ values (22.2, 27.8, and 33.3 s^-1^, respectively) than those of the wild-type form (*K*_m_ = 0.35 mM, *V*_max_ = 18.2 s^-1^) (Figure 4B). Therefore, we focused on these 3 mutants while selecting residues to mutate in the A and B subunits.

Our results suggested that when nonpolar leucine was substituted with polar asparagine (D(L^28^N), D(L^29^N), and D(L^170^N)), the ATPase activities of the mutants were almost the same as the activity of the wild-type complex (Table 1). Although these 2 amino acids with similar sizes differ in their hydrophobicity and polarity, they do not affect the activity and stability of V_1_-ATPase. The mutant in which the nonpolar/weakly hydrophobic alanine residue mutated to polar/weakly hydrophilic serine (D(A^169^S)) showed slightly lower ATPase activity and higher binding affinity for binding A_3_B_3_ than the wild-type (Table 1). However, changing basic/polar arginine residues (R^165^ and R^166^ of the D subunit) to neutral/nonpolar, helix-forming alanine [30] increased the ATPase activities of the mutants and lowered the binding affinities for A_3_B_3_; especially in the double RR^165-6^AA DF mutant (Table 1). We interpreted this to mean that the association/dissociation of the axis determines the ATPase activity level and possibly the rotational activity.

To measure the stability of the mutant catalytic domains, we used ATPase time-course assays. We allowed reactions to proceed for approximately 20 min and estimated the specific activity at 2-min intervals. The ATPase activities of all mutant catalytic domains after 15 min of the assay are listed in Tables 1 and 2. We observed that the specific activity of the mutants decreased continuously during the entire assay, with the A_3_B_3_D(RR^165-6^AA) F mutant showing the most rapid reduction in activity during the assay: Only 5% of the mutant’s original activity remained after 15 min (Figure 4C and Table 1); the activities of the A_3_B_3_D(R^165^A) F and A_3_B_3_D(R^166^A) F mutants decreased more slowly than that of the A_3_B_3_D(RR^165-6^AA) F mutant, but slightly faster than that observed with the wild-type protein.

**Table 2:** Summary of ATPase activities and binding affinities of V_1_ complexes containing mutant A_3_B_3_ and wild-type or mutant DF.

**Protein**	**(%**) of initial specific activity^*^	(**%**) of ATPase activity after 15 minutes of the assay^**^	***K*_D_ (nM**)** (using mutant A_3_B_3_ as ligand and wild-type or mutant DF as analyte**)
**Wild-type A_3_B_3_DF**	100	44	1.6 ± 0.1
**A(R^475^A**)**_3_B_3_DF**	186	29	1.0 ± 0.1
**A_3_B(V^388^A**)**_3_DF**	138	29	5.2 ± 1.1
**A_3_B(L^389^A**)**_3_DF**	214	32	10.9 ± 0.9
**A(R^475^A**)**_3_B_3_D(RR^165-6^AA**)**F**	193	12	14.8 ± 3.2
**A_3_B(V^388^A**)**_3_D(RR^165-6^AA**)**F**	191	15	10.8 ± 0.8

^*^See the Table 1 footnotes.

^**^Details presented in Table 1 footnotes.

ATPase activities of the reconstituted A_3_B_3_DF mutants were measured, and SPR assays were performed as described in Materials and Methods.

### Properties of the Mutated A_3_B_3_ Complexes

When V_1_-ATPase hydrolyzes ATP, the D subunit rotates inside the hexagonally arranged A_3_B_3_ complex and comes into contact with conserved residues of A and/or B subunits, which likely correspond to the conserved DELSEED loop of the β subunit of F-ATPase [19-21]. From structural and sequence analyses of *E* *. hirae* V-ATPase, we considered residues^480^DSLSDND^486^ of the A subunit to correspond to the DELSEED loop of F-ATPase. Furthermore, from the crystal structures of A_3_B_3_ and A_3_B_3_DF with or without the nucleotide AMP-PNP [15], we determined that residues R^475^, L^476^, and V^477^ of the A subunit and V^388^ and L^389^ of the B subunit were located near R^165^ and R^166^ of the D subunit. R^475^ of the A subunit appears to reside close to, but not in direct contact with R^165^ and R^166^ of the D subunit. Therefore, we mutated the residues present near the^480^DSLSDND^486^ sequence of the A subunit. We changed all the residues to alanine and found that we could still reconstitute the catalytic domains, A(R^475^A) _3_B_3_DF, A_3_B(V^388^A) _3_DF, and A_3_B(L^389^A) _3_DF, in the same manner as the wild-type (Figure 3) [14] with similar biochemical properties and stability in the presence of nucleotides [14]. In other cases, we could not reconstitute the catalytic domain as with the wild-type. The 3 mutant catalytic domains, A(R^475^A) _3_B_3_DF, A_3_B(V^388^A) _3_DF, and A_3_B(L^389^A) _3_DF, showed initial ATPase activities that were 1.9-, 1.4-, and 2.1-times higher, respectively, than the wild-type complex (Table 2). Thus the initial ATPase activity increased when arginine, a hydrophilic/large residue, or valine/leucine, strongly hydrophobic residues, were substituted with the weakly hydrophobic/nonpolar small amino acid alanine, which may be partially due to the stable helix-forming tendency [30]. Surprisingly, A(R^475^A) _3_B_3_ showed higher binding affinity (*K*_D_ = 1.0 nM) for the DF heterodimer than the wild-type complex (Table 2); we expected lower affinity than the wild-type because the mutant showed higher ATPase activity. We have not investigated this mutant further and the reason for this discrepancy remains unclear, although the conformational change caused by the R^475^A mutation may have strengthened binding with the DF axis in a way that is not relevant to the dissociation/association of the axis during rotation and, therefore, to ATPase activity. As expected, A_3_B(V^388^A)_3_ and A_3_B(L^389^A)_3_ showed lower binding affinities for DF (*K*_D_ values of 5.2 and 10.9 nM, respectively, Table 2).

The crystal structures of *E* *. hirae* V_1_-ATPase [15,16] show that residues R^475^ of the A subunit and V^388^ and L^389^ of the B subunit are located at the C-terminal regions near the RR^165-6^ residues of the D subunit. We expected strong interactions between these closely residing amino acids to influence ATPase activity. Moreover, the hydrophobic part of the arginine side chain can also possibly interact with the hydrophobic/nonpolar residues such as valine and leucine [31]. Thus, the substitution of these amino acids with the small amino acid alanine is expected to increase the ATPase activities by lowering binding affinities, which was the measured result with all mutants except the R^475^A mutant noted above (Figure 4D, Table 2). Recently, for yeast V-ATPase, “loosening” the V-ATPase complex was suggested to increase catalytic activity [32], and mutational studies on the ε-subunit of *E. coli* F-ATPase [25] showed that lowering binding affinity increased ATPase activity.

We once again used time-course experiments to determine the stability of the ATPase activities of these mutants (Figure 4D), and the results are summarized in Table 2. Specific activities decreased continuously during the assay for the mutants as with the wild-type and the D subunit mutants, and, after 15 min, the specific activities dropped to 29%, 29%, and 32% of initial activities for A(R^475^A) _3_B_3_DF, A_3_B(V^388^A) _3_DF, and A_3_B(L^389^A) _3_DF, respectively. All 3 mutants showed slightly higher reduction in activity than the wild-type complex, likely due to their lower binding affinities.

### Properties of the Reconstituted Double-Mutant A_3_B_3_DF Complexes

After constructing A, B, and D subunit mutants, we reconstituted double-mutant V_1_ domains with mutations in the D subunit and A or B subunit: A(R^475^A) _3_B_3_D(RR^165-6^AA) F and A_3_B(V^388^A) _3_D(RR^165-6^AA) F (Figure 3). We selected these combinations based on interactions of the mutants suggested by the crystal structures of *E* *. hirae* V_1_-ATPase [15,16]. Both double mutants showed higher initial ATPase activities (1.9 times for both A(R^475^A) _3_B_3_D(RR^165-6^AA) F and A_3_B(V^388^A) _3_D(RR^165-6^AA) F) and lower binding affinities than the wild-type (Table 2).

A(R^475^A) _3_B_3_D(RR^165-6^AA) F and A_3_B(V^388^A) _3_D(RR^165-6^AA) F showed remarkably rapid reduction in specific activities compared to the wild-type complex, but as with A_3_B_3_D(RR^165-6^AA) F, the original D subunit mutant, these 2 mutant complexes retained approximately 12% and 15% activities after 15 min (Table 2). To ensure that the low ATPase activity measured was not due to the substrate being depleted during the ATPase assay, we added excess NADH twice during the assay and found no noticeable change in activity (data not shown). We therefore speculate the activity decreased because of continuous dissociation of some amount of mutant DF from the A_3_B_3_ stator barrel during high-speed rotation.

ATPase activities and binding affinities exhibited reciprocal relationships in all mutants except A(R^475^A) _3_B_3_DF. These findings (Tables 1 and 2) indicate that the high ATPase activity, which likely depends on the rotation speed, results from the loose binding of the DF axis to the rotary ring A_3_B_3_.

### Wild-type A_3_B_3_DF (V_1_) Complex is an Optimized Rotary Motor

If close contact between 2 amino acids determines a protein’s function, a combined mutation of the amino acids may either produce a larger effect than a single mutation or compensate for the effect produced by a single mutation. The D(RR^165-6^AA) F mutant with A_3_B(V^388^A)_3_ showed higher ATPase activity with lower binding affinity than A_3_B(V^388^A) _3_DF, but nearly similar ATPase activity as A_3_B_3_D(RR^165-6^AA) F (Tables 1 and 2), which indicates that RR^165-6^ of the D subunit interacts with V^388^ of the B subunit in the “tight” form of the complex (A_CR_-B_CR_ pair, Figure 2B and G), as suggested by our crystal structures [15,16]. We consider this to be a compensation effect. Unexpectedly, the D(RR^165-6^AA) mutant with A(R^475^A) _3_B_3_ showed no change in ATPase activity or binding affinity (Tables 1 and 2) compared to D(RR^165-6^AA) with wild-type A_3_B_3_, indicating that R^475^ of the A subunit locating near RR^165-6^ of the D subunit does not contact directly but interacts functionally with RR^165-6^, again producing a compensation effect.

Our ATPase assay showed that the V_1_ domains were less stable when containing D(RR^165-6^AA) F, D(R^166^A) F, or D(R^165^A) F mutants (Figure 4C), which may be due to their lower binding affinities (Table 1). After 15 min, we found only 5% of the original ATPase activity for A_3_B_3_D(RR^165-6^AA) F (Figure 4C and Table 1) and 12% and 15% for A(R^475^A) _3_B_3_D(RR^165-6^AA) F and A_3_B(V^388^A) _3_D(RR^165-6^AA) F, respectively (Table 2). In contrast, wild-type and other DF mutants retained higher stability, possibly because of their higher binding affinities (Table 1). Moreover, incubation with ATP (especially under alkaline high-salt conditions), but not with ADP or AMP-PNP, stimulated the disassembly of the wild-type V_1_ complex (unpublished observation, part of a Ph. D. thesis of Arai S (2009): Study on the resolution and assembly of V_1_-ATPase from *Enterococcus* *hirae* . Tokyo University of Science, Japan). Therefore, we propose that tight binding of the DF axis is critical for stable ATPase activity. Considering its stability and rotation speed (activity), the wild-type A_3_B_3_DF (V_1_) complex appears to be a well-optimized rotary motor.
